# Colloidal Interactions of Microplastic Particles with Anionic Clays in Electrolyte Solutions

**DOI:** 10.1021/acs.langmuir.3c01700

**Published:** 2023-08-30

**Authors:** Dóra Takács, Tamás Szabó, Andrej Jamnik, Matija Tomšič, István Szilágyi

**Affiliations:** † MTA-SZTE Lendület Biocolloids Research Group, Interdisciplinary Excellence Centre, University of Szeged, Rerrich Bela ter 1, H-6720 Szeged, Hungary; ‡ Department of Physical Chemistry and Materials Science, University of Szeged, Rerrich Bela ter 1, H-6720 Szeged, Hungary; § Faculty of Chemistry and Chemical Technology, University of Ljubljana, Večna pot 113, SI-1000 Ljubljana, Slovenia

## Abstract

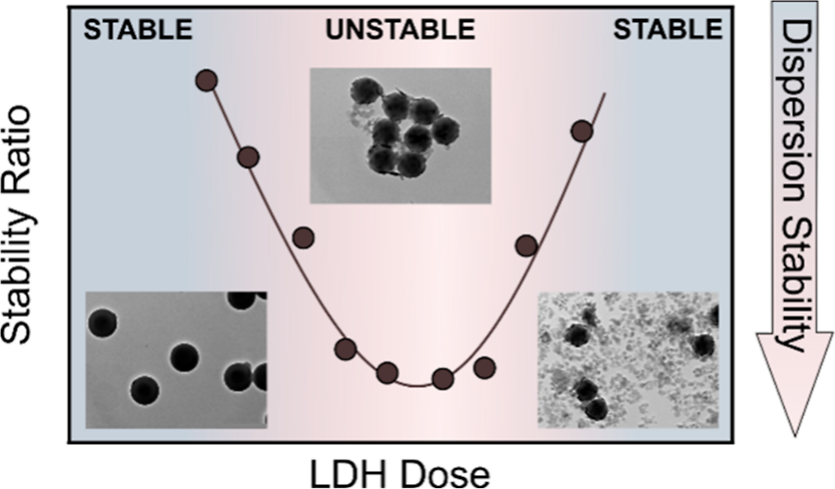

Homoaggregation of polystyrene microplastics (MPs) and heteroaggregation of MPs with anionic clay minerals, namely, layered double hydroxide (LDH), in different salt (NaCl, CaCl_2_, and Na_2_SO_4_) solutions were systematically investigated using light scattering techniques. The salt type and ionic strength had significant effects on the stability of both MPs and LDH particles individually and the results could be explained by DLVO theory and the Schulze–Hardy rule. However, once stable colloidal dispersions of the individual particles were mixed, heteroaggregation occurred between the oppositely charged MPs and LDH, which was also confirmed by transmission electron microscopy and X-ray scattering. Adsorption of the LDH particles resulted in neutralization and reversal of MPs surface charge at appropriate LDH doses. Once LDH adsorption neutralized the negative charges of the MP spheres, rapid aggregation was observed in the dispersions, whereas stable samples formed at high and low LDH concentrations. The governing interparticle interactions included repulsive electrical double-layer forces, as well as van der Waals and patch-charge attractions, the strength of which depended on the mass ratio of the interacting particles and the composition of the aqueous solvent. Our results shed light on the colloidal behavior of MPs in a complex aquatic environment and, in the long term, are also useful for developing LDH-based approaches for water remediation to remove contamination with MP particles.

## Introduction

The widespread presence of plastic waste, which is characterized by its high durability and resistance against chemical degradation, has led to a significant environmental problem.^1,2^ Regardless of the source of input, plastic debris in nature can break down into smaller pieces known as microplastics (MPs).^3^ These are plastic particles ranging in size from 5 mm to 100 nm that result from the fragmentation of larger plastic debris through a series of physicochemical processes in the environment, such as photodegradation, mechanical abrasion, and biodegradation.^4,5^

Due to their large surface area and functional groups on their surface,^6^ MPs can interact with a variety of components present in aqueous samples and thus serve as a carrier^7^ in the environmental matrix for problematic substances such as persistent organic pollutants,^8^ heavy metals^9,10^ and other contaminants with emerging concerns.^11,12^ The pathways and toxicity of the resulting composite materials are influenced by their stability and dispersibility,^13,14^ which can be affected by physicochemical processes such as aggregation, deposition, and resuspension.^15–17^ Therefore, several studies have investigated the effect of solution conditions on the homoaggregation behavior of MPs in water compartments, studying the effect of solution pH,^18^ temperature,^7^ electrolyte type, ionic strength,^19^ and macromolecules.^13,16,20^

However, once MPs enter the aquatic environment, they are inevitably susceptible to heteroaggregation with diverse minerals as the latter are abundant in soils and sediments. Hence, comprehensive investigations were conducted on the interactions between MPs and minerals,^21^ such as kaolin,^18^ iron oxide,^22,23^ gibbsite,^24^ and clay particles.^18^ In addition, heteroaggregation between MPs and other colloids was shown to alter the buoyancy of MPs and increase their sedimentation rate, which is a key process in efficient water treatment or during the migration of MPs in the environment.^25–28^

These phenomena, as well as the stability of the occurring particles, are further influenced by solution properties (e.g., pH, ionic strength, and natural organic matter content),^29^ as well as the features of the plastics (e.g., particle size, shape, and chemical composition) and other colloidal particles (e.g., composition, size distribution).^17,21,24,30,31^ Furthermore, the mass ratio and the surface charge of the interacting particles are also important, while the electrostatic forces have been proven to play a key role in the formation of heteroaggregates.^32–34^ Numerous studies have explored the impact of ionic strength on heteroaggregation, with emphasis on specific particle ratios.^35,36^ The critical coagulation concentration (CCC) for heteroaggregation (at a given particle mass ratio) has been identified as being highly sensitive to boundary conditions, especially when one of the particles approaches the charge reversal point. This sensitivity can be attributed to the interplay of double-layer forces between charged and neutral particles, which is highly influenced by the charge regulation characteristics of the weaker charged surface. Notably, when this surface is neutral, the charge regulation conditions play a decisive role in determining the sign of the interaction force. It is known that the interactions between MPs and other components determine the transport, fate, and ecological impacts of MPs,^37,38^ but the effects of certain solvent properties (e.g., presence of dissolved electrolytes of various compositions and valences) on their charging characteristics during heteroaggregation were not yet explored in detail. Therefore, further studies should be performed to unravel the origin of the interactions between MPs and minerals under various solution conditions.

**Scheme 1:**
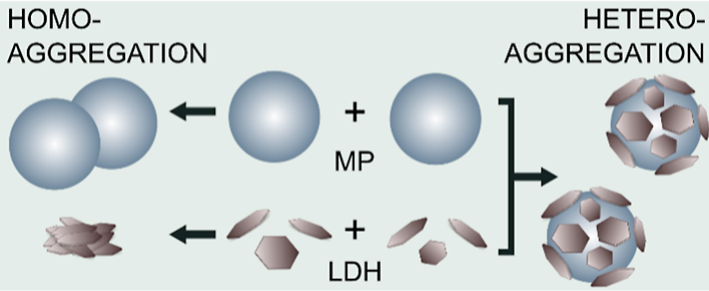
Visual Representation Depicting Homo- and Heteroaggregation Processes between MP and LDH Particles in Dispersions

In this study, homoaggregation of MPs and their heteroaggregation with anionic (possessing anion exchange capacity) layered double hydroxide (LDH) clay minerals (see Scheme 1) were systematically investigated using electron microscopy, light, and X-ray scattering techniques. Due to the wide range of applications of polystyrene plastics and since the majority of MPs display a negative surface charge in the environment, negatively charged sulfate modified polystyrene latex particle (PS) was selected as model MP particle,^15^ while LDH was chosen as a naturally occurring mineral (so-called hydrotalcite) that serves as an adsorption platform for negatively charged contaminants including plastics. The initial investigation focused on the colloidal behavior of the individual components (MP and LDH suspensions consisting of one type of particle). Charging and aggregation characteristics were investigated in various ionic environments by altering the composition and valence of the aqueous electrolyte solvent. Specifically, NaCl, CaCl_2_, and Na_2_SO_4_ were chosen due to their prevalence in natural waters and their ability to remain in a dissolved state under the specified experimental conditions to avoid precipitation issues. The obtained results were crucial for the subsequent heteroaggregation study, as an understanding of the stability of MP and LDH dispersions under the experimental conditions applied was necessary to accurately interpret the results obtained in the more complicated MP-LDH samples. While solution conditions, such as temperature and pH, undeniably exert a strong influence on the ongoing processes in nature, the present results still give unique information about the role of dissolved salts in the interparticle forces driving the plastic–clay interactions.

## Experimental Section

### Materials

Analytical grade salts, including sodium chloride (NaCl), sodium sulfate (Na_2_SO_4_), and calcium chloride (CaCl_2_) were purchased from VWR and used as received. All solutions were prepared using ultrapure water (Adrona) and the pH was adjusted to 9.0 with NaOH (AnalR NORMAPUR). To avoid dust contamination, all the salt stock solutions and the water were further filtered with a 0.1 μm syringe filter (Millex).

The negatively charged sulfate-modified polystyrene MPs were purchased from Thermo Fisher Scientific, with a mean diameter of 430 nm, a relative polydispersity of 1.8%, a solid content of 8.1% (w/v %), a specific surface area of 1.3 × 10^5^ cm^2^/g, and a charge density of −12 mC/m^2^. The LDH particles were synthesized using the flash coprecipitation method followed by hydrothermal treatment.^39–41^ In brief, a mixed metal ion solution was prepared by dissolving 0.2 M Mg(NO_3_)_2_ and 0.1 M Al(NO_3_)_3_ in water. The pH was adjusted to 10 using 4.0 M NaOH. The mixture was stirred under a N_2_ atmosphere for 30 min following centrifugation and washing steps. The resulting dispersion was transferred to an autoclave and treated at 120 °C for 24 h. After cooling, the slurry was filtered and dried at 50 °C overnight. For the experiments, stock samples were prepared by dispersing the solid LDH in water in calculated amounts.

### Electrophoresis

A Litesizer 500 instrument (Anton Paar) was used to quantify the electrophoretic mobility with a laser source operating at a wavelength of 658 nm and a scattering angle of 175°. The samples were prepared by mixing the appropriate amounts of salt solutions and water to obtain the desired electrolyte concentration. Next, the MP stock suspension was added to the samples, followed by the introduction of LDH particles into the stable MP suspensions. The LDH dose varied in the range of 0.01–50 mg/L, while the MP concentration (10 mg/L) and the final volume (2 mL) were kept constant in the experiments. During the investigation of homoaggregation, a concentration of 10 mg/L was utilized for both types of particles. The prepared samples were allowed to rest for 2 h at room temperature, after which the electrophoretic mobility of each sample was measured five times, and the average values were reported as final results.

To describe the surface charge of the particles, the electrophoretic mobilities were converted into electrokinetic potentials (ζ) using the Smoluchowski equation.^42^ Subsequently, the surface charge density at the slip plane was determined by fitting the potentials at different ionic strengths using the Debye–Hückel model as^43^
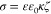
1where ε_0_ is the dielectric permittivity of the vacuum, ε is the dielectric constant of water, and κ is the inverse Debye length, which involves the contribution of all ionic species in the electrical double-layer.^42^

### Dynamic Light Scattering

Particle aggregation was followed by time-resolved dynamic light scattering measurements using a compact goniometer system (ALV/CGS-3) at a 90° scattering angle and borosilicate cuvettes (Kimble Chase). The correlation function was accumulated for 20 s and a second-order cumulant fit was performed to obtain the decay rate and subsequently to determine the hydrodynamic radius (*R*_h_).^44,45^ The change in the particle size was followed with time (*t*) under various experimental conditions, and the initial increase in *R*_h_ was used to calculate the apparent aggregation rate constant as follows^44^
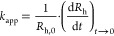
2where *R*_h,0_ is the initial hydrodynamic radius of the MP or LDH particle measured in a stable dispersion. The colloidal stability of the samples was expressed in terms of the stability ratio (*W*)
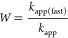
3where the fast subscript indicates fast or diffusion-controlled aggregation of the particles. In the heteroaggregation part, the value of *k*_app(fast)_ was determined in a 1 M NaCl solution.

The destabilization power of a given salt was quantified with the CCC, at which the transition from fast aggregation (*W* = 1) to stable dispersion (*W* ≫ 1) occurs, calculated using the following equation^46^
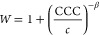
4where *c* is the molar concentration of the salt and the value of β was derived from the slope of the stability plots in the slow aggregation regime (i.e., before the CCC).

### Small-Angle X-ray Scattering

SAXS measurements were performed using a laboratory-modified old-Kratky type camera (Anton Paar) on a conventional X-ray generator (GE Inspection Technologies, SEIFERT ISO-DEBYEFLEX 3003; operating at 40 kV and 50 mA) equipped with focusing multilayer optics (Goebel mirror) and a block-collimation unit to provide a well-defined focused high-intensity Cu Kα line with a wavelength (λ) of 1.54 Å. Measurements were performed at 25 °C in a standard quartz capillary (outer diameter of 1 mm and wall thickness of 10 μm) and detected with a Mythen 1K microstrip solid-state diode-array detector (Dectris, Baden, Switzerland) in the range of the scattering vector (*q*) from 0.08 to 7 nm^–1^. The magnitude of *q* can be calculated as^47^
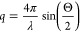
5where Θ is the scattering angle. The data were corrected for sample X-ray absorption and background scattering (obtained from water) and transformed to absolute scale using water as a secondary standard.^48^

### Solid State Characterization

To prove the formation of the LDH material powder, we collected X-ray diffraction (XRD) patterns with a Philips PW1830 diffractometer with Cu Kα (λ = 0.1542 nm) as a radiation source. The diffraction beam was detected over a 2Θ range of 5–80° with a step size of 0.02°. The morphology of the particles was examined by using transmission electron microscopy (TEM, FEI Tecnai G2). For TEM sample preparation, 5 μL of the particle dispersion was placed on a copper-coated carbon mesh, allowing it to adsorb for 10 s. The sample grids were prepared 30 min before the measurements.

## Results and Discussion

### Colloidal Characterization of MPs

Prior to exploring the heteroaggregation of MPs with LDHs, homoaggregation of MPs was investigated (see Scheme 1 to distinguish such homo- and heteroaggregation processes). The charging behaviors and colloidal stabilities of the individual particles were studied under different salinity in terms of concentration and ionic valence. In this way, the composition of the electrolytes was varied (NaCl, CaCl_2_, and Na_2_SO_4_) to assess the influence of the valence of cations and anions on the properties of the colloidal dispersions.

**Figure 1:**
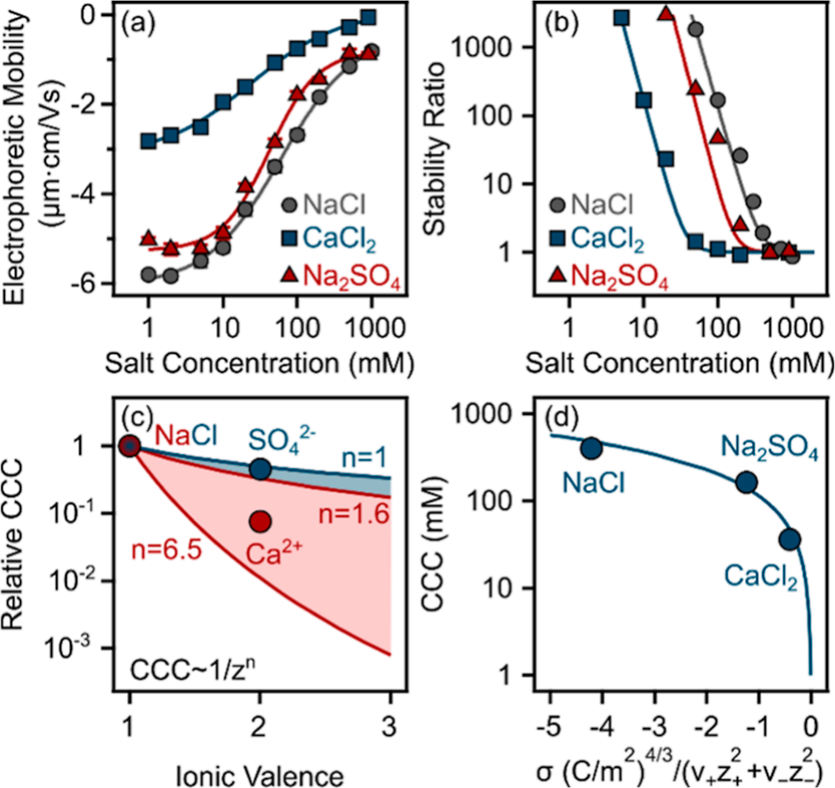
(a) Electrophoretic mobilities and (b) stability ratios of MPs as a function of the salt concentration adjusted with different electrolytes. The lines just serve to guide the eyes. (c) Relative CCC values (normalized to the CCC obtained in the presence of NaCl) as a function of the ionic valence. The solid lines indicate the direct (for *n* = 1.6 and 6.5 in eq 6) and the inverse (*n* = 1 in eq 6) Schulze–Hardy rules. (d) Dependence of the CCC on the charge density at the slip plane, which was normalized with the stoichiometry and the valence of the electrolytes. The solid line was calculated by eq 7.

The charging features of negative MP particles were followed by an electrophoretic light scattering technique. The results are depicted in Figure 1a. Accordingly, the absolute value of the MP particle mobilities decreased with the electrolyte concentration in each case due to charge screening by the ions and remained close to zero at higher ionic strengths. Although the MPs were negatively charged throughout the concentration range studied, the exact mobility values under a given experimental condition differed significantly due to specific ion adsorption. This was further confirmed by the charge density values, which were determined from the concentration-dependent mobility plots using eq 1, and they followed the NaCl > Na_2_SO_4_ > CaCl_2_ order, as can be seen from the obtained data gathered in Table 1.

**Table 1:** Characteristic Charging and Aggregation Data of MP and LDH Particles

particle	MP			LDH		
salt	NaCl	CaCl_2_	Na_2_SO_4_	NaCl	CaCl_2_	Na_2_SO_4_
σ (mC/m^2^)^a^	–27.8	–11.1	–25.4	8.4	21.0	0.04
CCC (mM)^b^	400.7	35.9	182.2	17.5	8.9	0.03
*k*_app(fast)_ (×10^–^^3^ s^–^^1^)^c^	0.23	0.22	0.24	1.17	1.09	1.17

a Surface charge density determined with eq 1.

b Critical coagulation concentration calculated by eq 4. The uncertainty of the CCC determination is about 10%.

c Apparent aggregation rate coefficient in the fast aggregation regime obtained by eq 2.

The aggregation processes were studied using the same experimental conditions (e.g., particle concentration, pH, salt concentration range, and composition) as those used for electrophoresis, enabling direct comparison of the observed trends. The results in Figure 1b show that the samples were stable at low electrolyte concentrations as indicated by the high stability ratio values, whereas at higher electrolyte concentrations, the dispersions became unstable. These two regimes are separated by the CCC, which parameter can adequately describe the destabilization power of the given salts, and the obtained values followed the order of NaCl > Na_2_SO_4_ > CaCl_2_, as shown in Table 1. These tendencies in the charging and aggregation features are typical for systems, in which the main interparticle forces originate from DLVO-type interactions such as van der Waals attraction and repulsion by the overlapping electrical double-layers.^49,50^

The tendency in the CCC values was further explored within the Schulze–Hardy rule,^51,52^ which implies that the CCC dependence on the ionic valence (*z*) can be quantified as
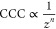
6where the exponent *n* depends on the surface charge and the hydrophobicity of the particles and the solvation level of the ions present in the solutions. Accordingly, for particles of low surface charge, the exponent is 1.6, while for highly charged particles, it is 6.5 when considering the valence of the counterions. These limits are referred to as the direct Schulze–Hardy rule.^52^ However, if one considers the effect of the valence of co-ions (same sign of charge as the particles) on the CCC, the dependence is much less significant and can be described with the inverse Schulze–Hardy rule (*n* = 1 in eq 6).^53^ In Figure 1c, the relative CCCs, i.e., CCSs normalized to the CCC obtained with NaCl electrolyte, and the CCC values expected from the direct and inverse Schulze–Hardy rule with the aforementioned limits are shown. The obtained results for the divalent counter (Ca^2+^) and co-ions (SO_4_^2–^) are in good quantitative agreement with the prediction of the rules, and the fact that the result for Ca^2+^ counterions appear between the limits indicates that the MP particles are moderately charged.

Subsequently, the aggregation mechanism was further investigated by plotting the experimental CCC values versus the calculated surface charge density data and comparing them to the CCCs calculated by the DLVO theory as^54^
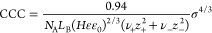
7where *N*_A_ is Avogadro’s number, *H* is the Hamaker constant, *L*_B_ is the Bjerrum length (0.72 nm at room temperature in water), ν_+_ and ν_–_ are the stoichiometric coefficients, and *z*_+_ and *z*_–_ represent the ionic valences for cations and anions, respectively.

To achieve the best agreement between the calculated and measured CCC data, a Hamaker constant of 3.7 × 10^–21^ J was used, as shown in eq 7. This value is well within the range reported earlier for polystyrene particles based on results from direct force measurements.^55^ The good agreement between the experimental and calculated data (Figure 1d) shows that the interparticle forces responsible for colloidal stability are predominantly of DLVO origin, arising from a combination of attractive van der Waals forces and repulsive electrical double-layer forces. However, ion-specific interactions play a significant role in determining surface charge densities and in influencing the strength of repulsive double-layer forces.

### Characterization of LDHs

**Figure 2:**
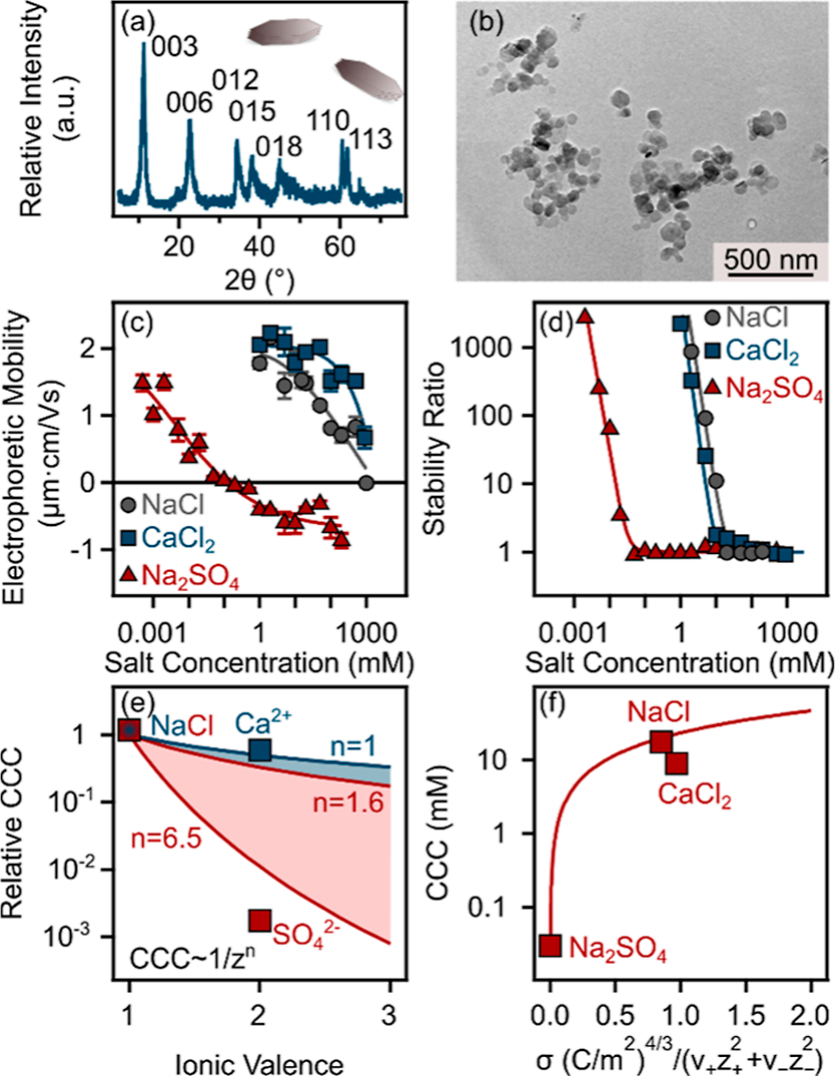
(a) Powder XRD pattern and (b) TEM image of LDH. (c) Electrophoretic mobilities and (d) stability ratios of LDH particles as a function of the salt concentration. The lines just serve to guide the eyes. (e) Relative CCC values (normalized to the CCC obtained in the presence of NaCl) as a function of the ionic valence. The solid lines indicate the direct (for *n* = 1.6 and 6.5 in eq 6) and the inverse (*n* = 1 in eq 6) Schulze–Hardy rules. (f) Dependence of the CCC on the charge density at the slip plane, which was normalized with the stoichiometry and the valence of the ions in the solution. The solid line was calculated with eq 7.

The successful synthesis of LDH was confirmed by XRD measurement prior to colloidal investigation. The obtained XRD pattern shown in Figure 2a reveals the crystal structure, which corresponds well to the standard diffraction pattern of LDH materials.^56^ In addition, the morphology of the LDH was visualized by TEM, which showed a typical hexagonal structure with some distortions, as can be seen in Figure 2b.

The colloidal characteristics of the LDH were investigated in a fashion similar to that in the case of MP particles. In Figure 2c one can see that LDH exhibits considerably high positive mobilities at low concentrations, which can be attributed to its structural charge. However, as the electrolyte concentration increases, the mobilities decrease and become nearly zero at high electrolyte concentrations, primarily due to the screening effect on the surface charge and the adsorption of anions onto the oppositely charged surface. The latter effect gave rise to slightly negative mobilities in the case of Na_2_SO_4_ at high concentrations, which phenomenon has already been reported for other LDHs in the presence of divalent anions.^57^ The obtained charge density values followed the order CaCl_2_ > NaCl > Na_2_SO_4_ (see Table 1).

Regarding homoaggregation of LDHs (see Scheme 1), the stability curves shown in Figure 2d exhibit the characteristic slow and fast aggregation regimes, like the MP systems discussed above. Nevertheless, for LDH platelets, the determined CCC values were substantially lower than for MP particles due to their lower surface charge density (data are shown in Table 1), and they decreased in the NaCl > CaCl_2_ > Na_2_SO_4_ order, which differs from the sequence obtained from the charge density data.

The obtained CCCs were compared to the prediction of the direct and inverse Schulze–Hardy rules in Figure 2e. For the SO_4_^2–^ ion, the experimental results show a stronger dependence than the calculated ones, indicating that it interacts with the oppositely charged surface specifically. The previously mentioned charge reversal and the remarkably low CCC value in the presence of SO_4_^2–^ ion further validate the high affinity of this ion to the LDH surface, which may originate from the weaker hydration of the anion and the possible formation of hydrogen bonds between the SO_4_^2–^ and the surface hydroxyl groups.^57,58^

The major interparticle forces between the LDH particles were also investigated by comparing the experimentally obtained and the calculated CCC data in Figure 2f (similarly as in the case of MP particles, and a Hamaker constant of 4.2 × 10^–20^ J was used in eq 7). The experimental data agreed relatively well with the calculated values for NaCl and Na_2_SO_4_, suggesting that their aggregation can be explained by the DLVO theory. However, the ion-specific interactions play an important role through the extent of the ion adsorption to the surface of LDH leading to different charge densities and thus, causing significant variation in the strength of the repulsive double-layer forces and subsequently, in the location of the CCCs.

Nevertheless, there is a clear deviation between the measured and calculated CCC values in CaCl_2_ solutions, as presented in Figure 2f. This observation suggests the contribution of additional (beyond van der Waals forces) attractive forces between the LDH particles in the presence of Ca^2+^ ions. Since the surface charge density of LDHs is higher in the presence of CaCl_2_ than in the case of NaCl (see Table 1), adsorption of the multivalent cation most likely took place on the like-charged surfaces. This result is in line with earlier findings obtained with positively charged colloidal particles and multivalent co-ions.^12^ Accordingly, the additional forces may originate from short-range attractions induced by the Ca^2+^-rich regions formed upon the adsorption of the divalent ions on the surface of the LDHs.

### Heteroaggregation between MPs and LDHs

After thorough colloidal characterization of the MP spheres and LDH platelets, the interactions between the oppositely charged particles were studied. In these experiments, the dose of LDH was systematically varied (the LDH dose corresponds to the mass of LDH added per 1 g of MP), while the MP concentration was kept constant in the samples (10 mg/L). The experiments were performed in the presence of NaCl, CaCl_2_, and Na_2_SO_4_ (to address the ion specificity) at three different electrolyte concentrations (to probe the electrostatic origin of the interparticle forces).

**Figure 3:**
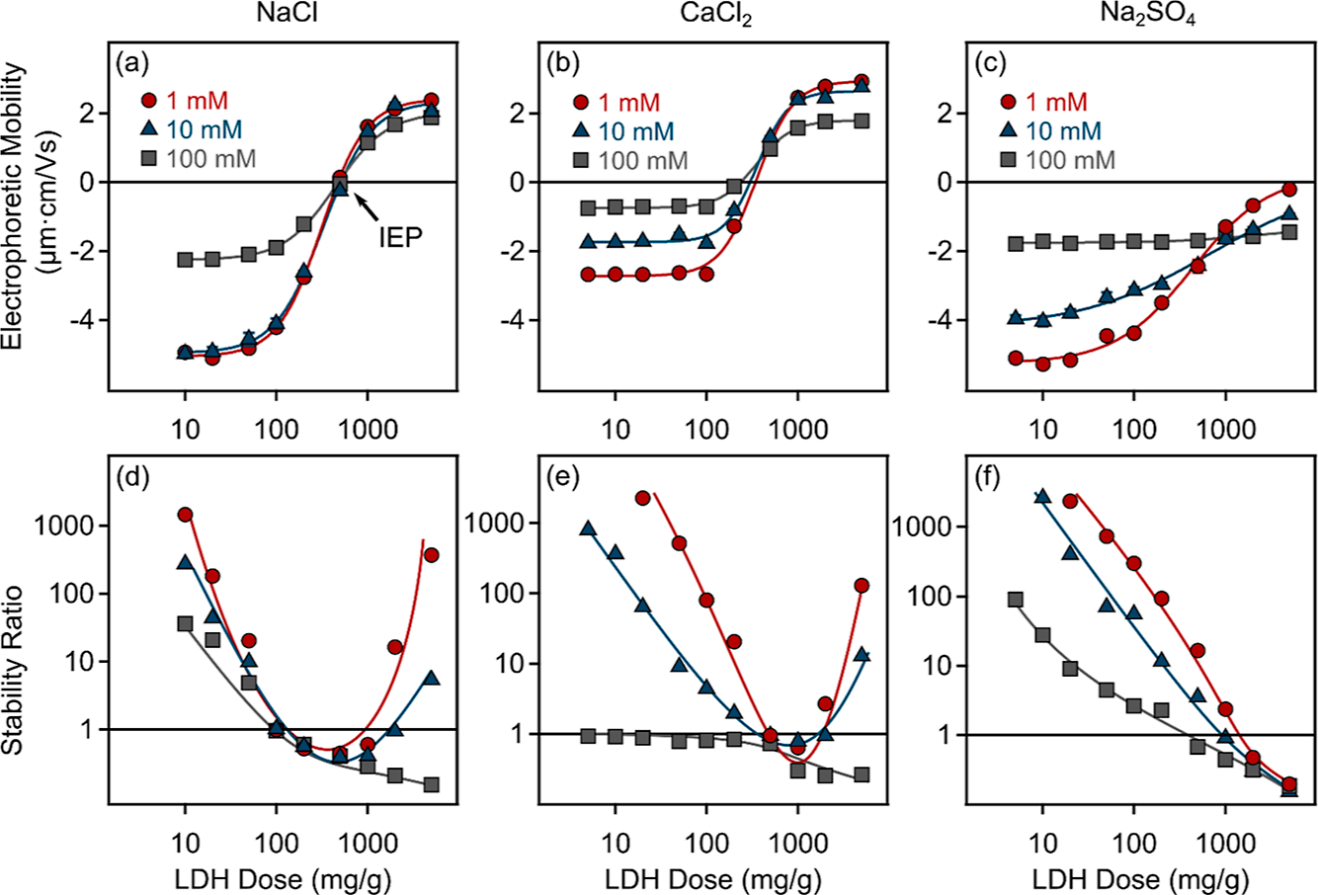
Electrophoretic mobility (a–c) and stability ratio (d–f) values of negatively charged MPs (10 mg/L) in the presence of LDH particles in the presence of NaCl (a,d), CaCl_2_ (b,e), and Na_2_SO_4_ (c,f) at different concentrations. The solid lines serve only to guide the eyes.

**Figure 4:**
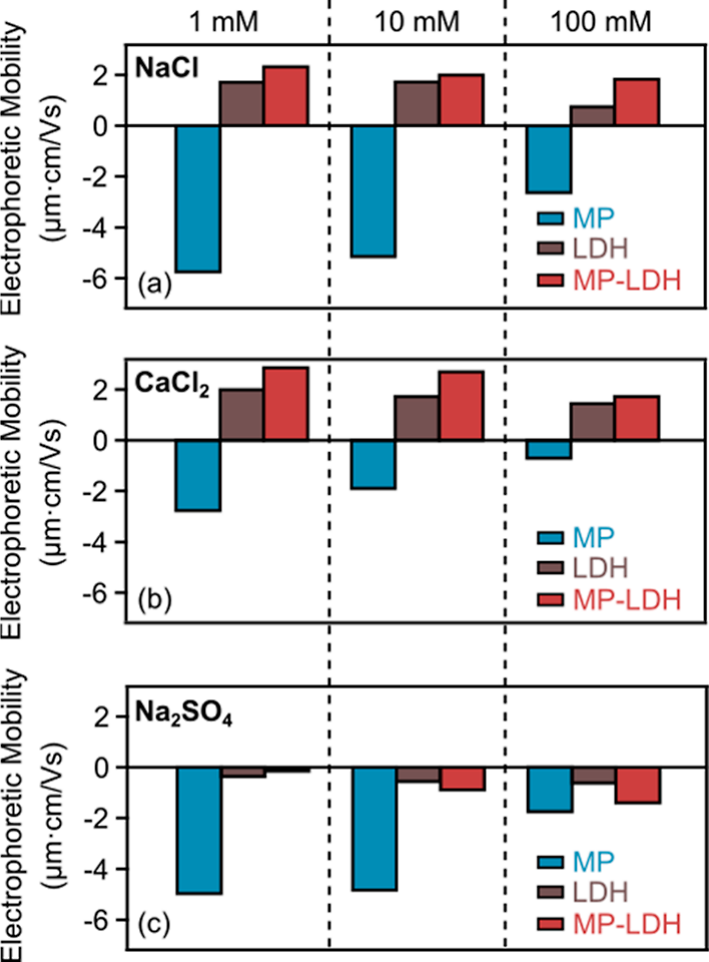
Electrophoretic mobility values of MP, LDH, and MP-LDH composite (at 5000 mg/g dose) in the presence of (a) NaCl, (b) CaCl_2_, and (c) Na_2_SO_4_ at different concentrations.

Surface charge characteristics assessed via electrophoretic mobility data are presented in Figures 3 and 4. Negative mobility values were observed at low LDH doses due to the negative charge of the MP spheres. The values measured in this regime were slightly higher than in the case of initial MP suspensions at the same electrolyte concentration and composition (see Figure 1a) because the LDH adsorbed on larger MP particles already at low concentrations due to the electrostatic interactions between the oppositely charged particles. In the case of NaCl and CaCl_2_ (see Figure 3a,b), charge reversal occurred with an increase in the LDH dose. Accordingly, mobility values increased with the increase in LDH concentration until the isoelectric point (IEP) was reached, at which the MP particles no longer exhibited overall net charge. Further adsorption beyond the IEP resulted in overcharging at higher LDH doses, and a plateau was reached at doses above 1000 mg/g. Beyond the onset of these plateaus, one presumes that any additional LDH introduced remained dispersed in the bulk. Such a change in the sign of the surface charge is typical for colloids when they are present together with oppositely charged polyelectrolytes^59,60^ or mineral particles.^32,61^ In contrast, the negative charge of the LDH particles at Na_2_SO_4_ concentrations higher than 0.1 mM (see Figure 2c) prevented any charge reversal of MP in the presence of Na_2_SO_4_ (see Figure 3c), and thus no IEP could be determined.

Furthermore, the changes that occurred in the mobilities with variations in electrolyte concentration were also analyzed and compared. When the salt level increased, the qualitative behavior remained overall very similar, i.e., the magnitude of mobilities was reduced due to the charge screening by salt constituents. This reduction was more profound at low LDH doses, reflecting the more effective screening of the MP surface charge by counterions, which leads to lower electrophoretic mobilities. In contrast, such a screening was not that efficient at high LDH doses, where the MP particle surface was saturated with LDHs. These differences in the tendencies in mobilities at low and high LDH concentrations are likely due to the higher surface charge of the MP, which is also reflected by the higher magnitude of the mobility values compared to LDHs. The correlation between the mobility data and the type or concentration of the salts in MP, LDH, and MP-LDH dispersions is shown in Figure 4.

Accordingly, in NaCl solutions (see Figure 4a), the decrease in the magnitude of the electrophoretic mobility of MP is steeper than for the systems containing also LDH. This difference is less striking in the presence of CaCl_2_ (Figure 4b) due to the adsorption of the Ca^2+^ ions on both type of particles, as discussed above. For Na_2_SO_4_, however, the absolute mobility values decreased for MP, while they increased for LDH and MP-LDH by increasing ionic strength (Figure 4c). This is due to the charge reversal process, which progressively elevates the negative charge of LDH particles owing to SO_4_^2–^ adsorption.

In addition, in the case of NaCl, the IEP was approximately the same regardless of the electrolyte concentration, suggesting that the role of coadsorbing ions is weak. In contrast, for CaCl_2_, there was a slight decrease in the IEP with the increase in electrolyte concentration, meaning that a smaller amount of LDH was needed to achieve charge neutralization. This indicates that the Ca^2+^ ions and LDHs compete for MP’s adsorption sites. These findings prove that the electrostatic interactions between the particles indeed play a major role in the adsorption mechanism.

The aggregation properties were also assessed while the LDH dose was varied in time-resolved DLS measurements. In the case of NaCl (Figure 3d) and CaCl_2_ (Figure 3e) at low salt levels, the stability plots exhibit the characteristic U-shapes corresponding to charge reversal, while this U-shape trend in the data can be qualitatively explained by the DLVO theory.^60,61^ Accordingly, the aggregation near the IEP is rapid due to attractive van der Waals forces, which are the dominant forces between neutral particles. When moving away from the IEP in either direction, the magnitude of the surface charge increases, leading to increasingly stronger repulsion due to the overlap of diffuse layers and thus causing higher stability ratios. A similar trend in colloidal stability was observed in other systems containing oppositely charged colloidal particles.^61,62^ However, in the presence of Na_2_SO_4_ (Figure 3f), no restabilization occurred at any electrolyte concentration even at high LDH doses since no charge reversal took place (see Figure 3c) and thus no stabilizing forces were present.

The change in ionic strength affects the stability ratios significantly since the relatively narrow U-shaped curves, obtained at 1 mM NaCl concentration, widen at 10 mM and become almost open at 100 mM salinity. This tendency can be explained by the reduced electrostatic repulsion between particles due to the increased charge screening at higher salt concentrations.^60^ Although the DLVO theory qualitatively describes the observed tendency, the stability ratios below one in the intermediate LDH doses suggest that additional attractive forces must be present in addition to the classical van der Waals dispersion forces. One plausible explanation may be that this attraction arises from lateral charge inhomogeneities that occur when LDH platelets adsorb to the oppositely charged MP surface in line with earlier results reported in similar systems.^61^ The adsorbed LDHs form positively charged patches on the MP and Coulomb attraction takes place when another MP particle with negatively charged vacancies on its surface approaches. This electrostatic interaction leads to an acceleration of aggregation, resulting in higher apparent aggregation rate coefficients than the values determined for pure MP suspensions at high salt content, in which only van der Waals attraction is present. In addition, once MPs and clays are similarly charged (e.g., after IEP and in the presence of Na_2_SO_4_), the interactions between them can be notably influenced by depletion interactions.^63,64^ These are entropic forces that emerge when smaller particles, such as polymers or colloids, are present in a solution in considerably high concentrations. When larger particles are introduced into the same system, the smaller particles can be excluded from the region between the larger ones causing a lower concentration or “depleted” region around them. As a result, an additional attractive force between the larger particles occurs, resulting in accelerated aggregation (*W* ≪ 1). Indeed, the apparent aggregation rates in this regime during heteroaggregation of MP and LDH particles were found to vary between (0.35–1.49) × 10^–3^ s^–1^ depending on the electrolyte concentration and composition, which data are significantly higher than the one measured for MP in 1 M electrolyte solutions in the absence of LDH (Table 1). It is assumed that the joint effect of attractive patch-charge and depletion forces is responsible for this increase in the heteroaggregation rates; however, further investigation is necessary to gain a comprehensive understanding of the underlying processes in this regime.

**Figure 5:**
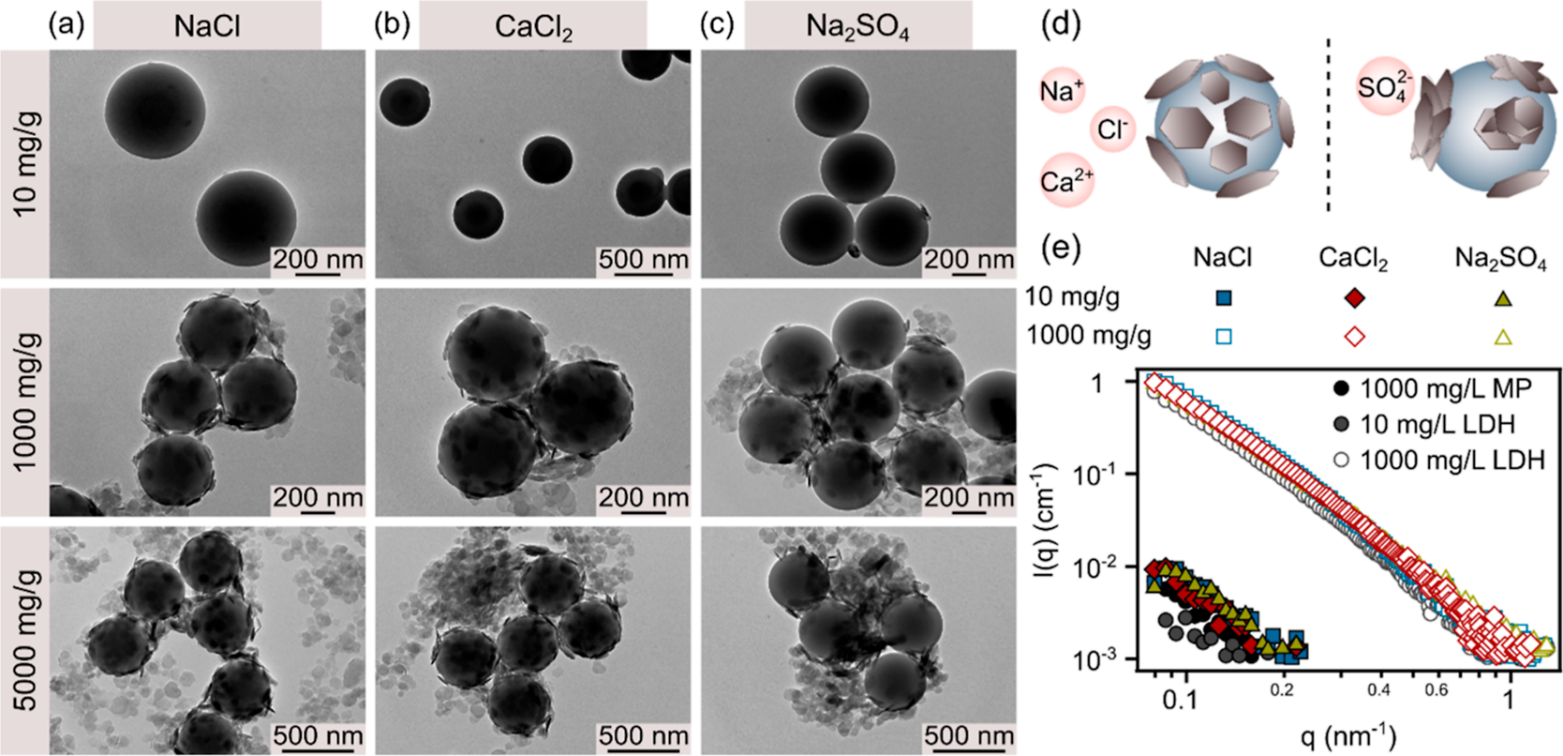
TEM images of the MP particles in the presence of different LDH doses and (a) NaCl, (b) CaCl_2_, and (c) Na_2_SO_4_. Ten mg/g refers to a low dose, 1000 mg/g is near the IEP, and 5000 mg/g corresponds to a dose, where the MP underwent charge inversion. (d) Schematic representation of the ion-specific effect on the morphology of heteroaggregates. (e) The experimental SAXS curves of MP, LDH, and MP-LDH dispersions at different concentrations. The salt concentration was 1 mM in all samples.

The lowest stability ratio values were obtained in the presence of Na_2_SO_4_. This can be explained as the LDHs tend to aggregate even at a very low Na_2_SO_4_ concentration (Table 1), and thus, they are adsorbed in aggregated form onto the MP surface, resulting in more pronounced patches, which causes stronger electrostatic interactions between the composite particles. This fact was confirmed by the TEM measurements evidencing that the surface coverage of MP in the presence of NaCl (Figure 5a) and CaCl_2_ (Figure 5b) was more homogeneous compared to the Na_2_SO_4_ (Figure 5c) case, where broader empty spaces could be observed on the MP surface at higher LDH doses due to the presence of adsorbed LDH aggregates (Figure 5d). TEM measurements were carried out at three different LDH doses, where 10 mg/g refers to a low dose, 1000 mg/g is near the IEP and 5000 mg/g corresponds to a dose, where the MP underwent charge inversion. The TEM images proved that when the electrophoretic mobility curves reach an adsorption saturation plateau (above 1000 mg/g doses), the LDH particles, which were further added to the system, remained in solution; thus, they can be seen separately from the MP.

Although the total size of the MP and LDH particles is well above the experimental resolution of the SAXS method, SAXS curves of the low and medium dose samples could still be measured. It was expected that one could detect differences in the scattering curves due to the different ion-specific surface phenomena in these samples. The resulting experimental SAXS scattering curves are shown in Figure 5e, where the SAXS data for pure MP and LDH particles in water at the appropriate concentrations are also shown for reference. Unfortunately, it turned out that the SAXS method was not sensitive enough to detect the ion-specific effects in these samples, most likely due to the insufficient concentration of the scattering particles and consequently to the too-weak scattering signal.

As the reference samples show, for both low and medium LDH dose samples, most of the signal comes from LDH particles, while MPs contribute only slightly. Nevertheless, the dispersions containing MP and LDH particles in electrolyte solutions all show an increased scattering signal compared with the reference samples, clearly indicating the adsorption of LDH on the surface of the MP particles and confirming heteroaggregation in these samples. Namely, pure aqueous electrolyte solutions showed no “excess scattering” compared to the scattering of pure water, which was used as “background scattering” and subtracted from the raw SAXS data.

All of these observations prove that the concentration and composition of the electrolyte affect not only the charge and aggregation characteristics of the individual and heteroaggregated particles but also the morphology of the resulting composite particles.

## Conclusions

This study systematically investigated the homoaggregation of polystyrene MPs and LDH as well as the heteroaggregation of MP with LDH in various salt solutions (NaCl, CaCl_2_, and Na_2_SO_4_). The stability of the individual particle systems was affected by the type and concentration of the electrolyte, which could be explained by the DLVO theory and the Schulze–Hardy rule. Regarding heteroaggregation processes, it was found that the mass ratio of LDH and MP is a critical parameter controlling the charging and aggregation features. Accordingly, electrostatic attraction between the negatively charged MPs and positively charged LDHs resulted in charge neutralization and subsequent overcharge at sufficiently high doses of LDH in NaCl and CaCl_2_ solutions. The aggregation rates increased near the IEP and stable suspensions were observed away from this point, where the particles possess sufficient surface charge for electrostatic stabilization. The predominant interparticle forces were found to be repulsive electric double-layer and attractive van der Waals forces of DLVO type, while near the IEP, an additional attractive force, known as the patch-charge attraction, was also found owing to the LDH patches formed on the surface of MP upon adsorption. These interactions were significantly affected by the amount of LDH particles adsorbed on the surface of MP and by the type and concentration of the background electrolyte. In addition, the morphology of the resulting composite particles was also influenced by the ionic environment. When the salt concentration exceeded the CCC value of the individual particles, a significant and observable change in morphology occurred. Our results suggest that the variability of environmental conditions strongly influences the charging and aggregation properties of MPs, which in turn affects their fate and transport in natural waters. In addition, understanding the effects of different ionic compounds on heteroaggregation is critical for interpreting the environmental behavior of MPs, when they coexist with natural colloids. Based on the present findings and knowing the physicochemical characteristics of the particles under different environmental conditions, the charging features and stability regimes can be qualitatively predicted in MP-clay systems. These results may also make an important contribution to the development of LDH-based approaches in water remediation to eliminate MP contamination.
